# Dynamics of Low-Level Viremia and Immune Activation after Switching to a Darunavir-Based Regimen

**DOI:** 10.3390/v16020182

**Published:** 2024-01-25

**Authors:** Arjen J. Stam, Ninée V. E. J. Buchholtz, Wouter F. W. Bierman, Reinout van Crevel, Andy I. M. Hoepelman, Mark A. A. Claassen, Heidi S. M. Ammerlaan, Berend J. van Welzen, Marjo E. E. van Kasteren, Steven F. L. van Lelyveld, Dorien de Jong, Kiki Tesselaar, Matthijs van Luin, Monique Nijhuis, Annemarie M. J. Wensing, LOWERIT Study Team

**Affiliations:** 1 Translational Virology Research Group, Department of Medical Microbiology, University Medical Center Utrecht, 3584 CX Utrecht, The Netherlands; 2 Department of Internal Medicine and Infectious Diseases, University Medical Center Utrecht, 3584 CX Utrecht, The Netherlands; 3 Department of Infectious Diseases, Public Health Service Amsterdam, 1018 WT Amsterdam, The Netherlands; 4 Department of Internal Medicine, University Medical Center Groningen, University of Groningen, 9713 GZ Groningen, The Netherlands; 5 Department of Internal Medicine, Radboud University Medical Center, 6525 GA Nijmegen, The Netherlands; 6 Department of Internal Medicine, Rijnstate Hospital, 6815 AD Arnhem, The Netherlands; 7 Department of Internal Medicine, Catharina Hospital, 5623 EJ Eindhoven, The Netherlands; 8 Department of Internal Medicine, ETZ Hospital, 5022 GC Tilburg, The Netherlands; 9 Department of Internal Medicine, Spaarne Gasthuis, 2000 TM Hoofddorp, The Netherlands; 10 Department of Immunology, University Medical Center Utrecht, 3584 CX Utrecht, The Netherlands; 11 Department of Clinical Pharmacy, University Medical Center Utrecht, 3584 CX Utrecht, The Netherlands

**Keywords:** HIV-1, low-level viremia, immune activation, HIV pathogenesis, replication, production

## Abstract

There is an ongoing debate regarding whether low-level viremia (LLV), in particular persistent LLV, during HIV treatment with optimal adherence originates from low-level viral replication, viral production, or both. We performed an observational study in 30 individuals with LLV who switched to a boosted darunavir (DRV)-based therapy. In-depth virological analyses were used to characterize the viral population and the (activity) of the viral reservoir. Immune activation was examined using cell-bound and soluble markers. The primary outcome was defined as the effect on HIV-RNA and was categorized by responders (<50 cp/mL) or non-responders (>50 cp/mL). At week 24, 53% of the individuals were considered responders, 40% non-responders, and 7% could not be assigned. Sequencing showed no evolution or selection of drug resistance in the non-responders. Production of defective virus with mutations in either the protease (D25N) or RT active site contributed to persistent LLV in two individuals. We show that in about half of the study participants, the switch to a DRV-based regimen resulted in a viral response indicative of ongoing low-level viral replication as the cause of LLV before the switch. Our data confirm that in clinical management, high genetic barrier drugs like DRV are a safe choice, irrespective of the source of LLV.

## 1. Introduction

A minority of people with HIV (PWH) demonstrate low-level viremia (LLV) despite adequate adherence to antiretroviral therapy (ART) [1]. The presence of LLV creates uncertainty about the effectiveness of therapy and is considered a risk factor for subsequent virological failure [2,3,4,5,6]. Guidelines differ in their recommendations on the management of LLV, but usually include additional follow-up visits, adherence counseling, HIV-RNA measurements, drug resistance testing, or a change in ART [7]. When ART is adapted because of LLV, a switch to a regimen containing a high genetic barrier antiretroviral drug such as darunavir (DRV) can be considered [8,9].

The debate about the origins of LLV is ongoing. Evidence is available that LLV can be a result of both viral production [10] as well as viral replication [11] and originating from cellular or anatomical reservoirs [12]. Although suboptimal adherence can cause blips [13] or LLV [14], it is not in all circumstances an explanation for LLV. Further complicating this debate is that there is not yet a universally accepted definition of LLV, and it can include viral loads between 50 and 1000 cp/mL (WHO definition) or viral loads only between 50 and 200 cp/mL (DHHS/IAS definition), with a further subdivision in persistent and transient LLV [4].

It is important to distinguish between viral production and ongoing replication on an individual level, as the latter could lead to ART failure and drug resistance [15]. For individuals with blips and transient LLV, issues with adherence resulting in replication may be more likely than in those with persistent LLV [4,13,16]. Individuals presenting with higher loads (range 400–999 cp/mL) are more at risk for ongoing replication and subsequent virological failure than those with lower-range viral loads (50–399 cp/mL) [6]. Comparably, blips with a higher magnitude (>500 cp/mL) are more at risk for viral rebound compared to lower-range blips [17]. In addition, continuous viral production and/or replication fuels persistent inflammation, activation, and immune dysfunction, which will in turn contribute to viral production and proliferation of infected cells, reinforcing this vicious cycle [18,19,20].

During ART, the majority of proviruses in the reservoir are defective and therefore unable to replicate, with only approximately 2% of the proviral DNA being replication competent [21]. A correlation between the size of the proviral reservoir and the presence of LLV has been suggested [22]. However, it is unknown whether the activity and the composition of the viral reservoir is related to LLV. To measure the size of the reservoir and whether the proviral DNA is intact, a high-throughput digital droplet PCR (ddPCR) called the intact proviral DNA assay (IPDA) has been developed [21]. Together with an assay that identifies cell-associated multiply spliced RNA (msRNA) indicative of the production of RNA transcripts, it is possible to accurately quantify the size and activity of the HIV reservoir.

Our primary objective is to observe the effects on HIV-RNA load by switching to a boosted DRV-containing regimen. We anticipate that LLV due to ongoing replication will recede after the switch. We hypothesize that even if replication did continue in certain anatomical compartments, the likelihood of selection of resistance will be low due to the switch to a high genetic barrier drug. Additionally, we evaluated the effects on viral evolution, the viral reservoir, and the dynamics of immune activation.

## 2. Materials and Methods

### 2.1. Study Population

A total of 30 individuals of 18 years and older participated in this multicenter observational study of 48 weeks (primary endpoint at 24 weeks) in 7 specialized HIV care centers across the Netherlands from December 2014 until December 2018, with an additional follow-up of HIV-RNA data until 2022. The study was approved by the Institutional Review Board (IRB) of University Medical Center Utrecht (14-018). Individuals who switched to a boosted DRV-containing regimen (once daily 800 mg or twice daily 600 mg) while maintaining the nucleotide/nucleoside reverse transcriptase inhibitor (NRTI) backbone could be included. Any switch from a non-nucleoside reverse transcriptase inhibitor (NNRTI), protease inhibitor (PI), or an integrase strand transfer inhibitor (INSTI) to boosted DRV was allowed. Past use of DRV in a previous regimen was allowed, but not when it was used at screening. A strict definition of LLV was chosen in which only individuals who were using ART for at least 48 weeks and demonstrated LLV despite adequate antiviral therapy and adherence counseling were included. LLV was defined as ≥2 viral loads (VL) between 50 and 1000 cp/mL within a year. In between two measurements of 50–1000 cp/mL, a viral load below 50 cp/mL was allowed if residual viremia was present, but not when there was no target detected (0 cp/mL). Additionally, individuals had to use at least 48 weeks of ART, and after the initiation of therapy, at least one viral load had to be below 200 cp/mL.

A blip was defined as an isolated viral load of 50–499 copies/mL between measurements of <50 copies/mL within a year. Individuals were excluded if they had major protease inhibitor mutations for DRV (I47V; I50V; I54M/L; L76V; I84V) [23], clinical signs of an opportunistic infection, or a high likelihood of poor therapy adherence.

### 2.2. Collection of Data and Samples

Blood and clinical data were collected at baseline, week 4, week 12, week 24, and week 48 after the switch to a boosted DRV-containing regimen. Additional data on HIV-RNA and ART use were available in the years following the 48-week study period from a subset of participants who gave consent for use of data after the primary study period. Isolation of peripheral blood mononuclear cells (PBMCs) was performed using Ficoll-Paque™ (Fisher Scientific, Landsmeer, the Netherlands) and a Leucosep^®^ (Greiner Bio-one, Alphen aan den Rijn, the Netherlands) tube. PBMCs were pelleted via centrifugation and counted using the Beckman Coulter Cell counter (Woerden, The Netherlands). Isolated cells were stored in a mixture of fetal bovine serum (FBS), Iscove’s Modified Dulbecco’s Medium (IMDM) and dimethyl sulphate oxide (DMSO) at −135 °C.

### 2.3. HIV-RNA Load and Virological Response

HIV-RNA load in plasma was measured using a COBAS AmpliPrep/COBAS Taqman HIV-1 assay or the Abbott RealTime HIV-1 Viral Load assay depending on the participating site. The primary endpoint at week 24 was defined as a short-term virological response and categorized by responders (VL < 50 cp/mL) or non-responders (VL > 50 cp/mL). If no data at week 24 were available, the closest time point to the primary endpoint at week 24 was taken to assign the short-term response category. In a post hoc analysis, additional follow-up data were used to assign a long-term virological response category of persistent responders (VL < 50 cp/mL in all measurements), individuals with persistent LLV (VL > 50 cp/mL in more than two measurements, >25% of measurements) or individuals with 1–2 blips. Plasma RNA was isolated according to an LLV protocol using higher plasma inputs varying from 1.0 to 6.0 mL depending on the VL, followed by ultracentrifugation, then RT-PCR and population-based Sanger sequencing of reverse transcriptase and protease (pol). RNA sequences and the presence of any type of mutation was evaluated using the Stanford HIVdb-algorithm version 8.5. The evolution of the viral population was evaluated using MEGA-X version 10.0.1 and using the dN/dS ratio with the Nei-Gojobori model when two or more pol sequences from one individual were available.

### 2.4. Total and Intact/Defective HIV-1 DNA Quantification

Genomic DNA was isolated using the dNeasy Blood and tissue kit (Qiagen, Venlo, The Netherlands), according to the manufacturer’s guidelines. A single fluorescent ddPCR was used to target the LTR [24]. An intact proviral DNA assay was used to quantify HIV-1-intact and defective DNA at the baseline and week 24. This multiplex ddPCR targets both the Ψ (psi) region and part of the Env region and was optimized to correctly quantify subtype B and C [25]. Intact proviral DNA could not be measured for other subtypes found in this study, such as CRF02_AG, F, and A. The DNA shearing Index (DSI), as well as the total number of copies/1 × 10^6^ PBMCs, was computed using the RPP30 household gene [21]. As a positive control, Jlat 15.4 cells (NIH AIDS Reagent Program, 9848) were used. DNA from PBMCs of HIV-negative donors was used as a DNA template control, and water was used as a no template control. The cycle conditions were defined according to the manufacturer’s protocol (Biorad) [26]. The results were analyzed using Quantasaft version 1.7.4. Samples were excluded if they did not meet the cutoff of at least 100,000 cells measured or if less than 7 copies were detected in the psi signal, 7 copies in the env signal, and 6 copies in the double fluorescent signal.

### 2.5. msRNA Quantification

Cell-associated RNA was isolated using the Rneasy mini kit (Qiagen) according to the manufacturer’s guideline from samples at baseline and week 24. HIV-1 RNA was quantified using the ddPCR targeting the msRNA region (see Supplementary S1). Isolated RNA was converted to complementary DNA (cDNA) with a gene-specific reverse primer and the Taqman Reverse Transcription Reagents (Invitrogen, Fisher Scientific, Landsmeer, the Netherlands). The ddPCR was performed according to the manufacturer’s protocol (Biorad, Veenendaal, The Netherlands). As a positive control, gblocks designed for msRNA subtype B and subtype C were used. RNA isolated from PBMCs of HIV-negative donors was converted to cDNA and used as a template control, and water was used as a no template control. The cycling conditions were defined according to manufacturer’s protocol, except for an adaptation in the annealing temperature from 60 °C to 58 °C. Analysis of the results was performed using Quantasaft version 1.7.4.

### 2.6. Immune Activation

Cell-bound markers were evaluated at baseline and week 24 using flow cytometry. Briefly, cryopreserved PBMCs were thawed in RPMI 20% FCS, washed using PBS, and incubated with mouse monoclonal antibodies against CD4 (fluorophore BV510; clone RPA-t4; Biolegend, San Diego, CA, USA), CD8 (fluorophore PercP-CY5.5; clone SK-1; BD Pharmingen, Franklin Lakes, NJ, USA), CD38 (fluorophore APC; clone HIT-2; eBioscience, Thermo Fisher Scientific, Waltham, MA, USA), and HLA-DR (fluorophore BV711; clone G46-6; Biolegend, San Diego, CA, USA). Fluorescence minus one (FMO) controls were used to define positive gates for the expression of different proteins. The analysis was performed using BD FACS Diva 9.0.1. For soluble markers, a multiplex immunoassay was used, as previously described [27,28]. In short, aspecific heterophilic immunoglobulins (IL-6, IL-1b, IP-10, MCP-1 MIP-1a, MIP-1b, sICAM-1, sCD14, sCD163, MIG) were pre-absorbed with HeteroBlock (Omega Chemicals, Hebron, IN, USA). Measurements were performed using a Bio-Rad FlexMAP3D in combination with xPONENT software version 4.1 (Luminex, Austin, TA, USA). Data analysis was performed using Bioplex Manager 6.1.1 (BIO-RAD).

### 2.7. DRV Drug Concentrations

Plasma concentrations of DRV, taken at week 4 and 24 after switching to DRV, were analyzed at the Department of Clinical Pharmacy of the UMC Utrecht using a validated liquid chromatography–mass spectrometry (LC-MS/MS) method. The calibration curve was linear over a concentration range of 0.0732 to 8.06 mg/L. The accuracy values for DRV were 98%, 98%, and 99% at 0.0732, 0.758, and 2.88 mg/L, respectively. At the same concentrations, the precision values (between day, coefficient of variation) were 9.8%, 3.6%, and 3.4%, respectively. Self-reported adherence was assessed using a Dutch version of the modified medication adherence self-report inventory (MMASRI).

### 2.8. Data Analysis and Statistics

Descriptive statistics were used for most of the parameters. The participants were assigned to a group of responders (<50 cp/mL) or non-responders (≥50 cp/mL) for (short-term) virological response at the primary endpoint (week 24). The categories for long-term response in the post hoc analysis included persistent responders, individuals with persistent LLV, and individuals with 1–2 blips. The Mann–Whitney U test was used when comparing variables between responders and non-responders, and the Kruskal–Wallis test was used when a third response category was included in the comparison. The Wilcoxon-signed rank test was used for comparing other parameters between the baseline and week 24. The chi-square test was used to test for associations between categorical data. Correlations were determined using a Spearman rank correlation. All the data were analyzed using SPSS Statistics version 29.0.1. A confidence interval of 95% was used, and *p* < 0.05 was defined as significant. A Bonferroni adjustment was performed to correct for multiple testing for immunological parameters. A principal component (factor) analysis was performed to identify the variables that explained most of the variance. The figures were made using SPSS Statistics version 29.0.1 or GraphPad Prism (v.10.1.0).

## 3. Results

The study population consisted of 30 individuals with a baseline a median age of 49 years old, a mean viral load of 153 cp/mL, and 86.7% were male. The other baseline characteristics are shown in Table 1. ART regimens consisting of a tenofovir (TDF/TAF) and an emtricitabine (FTC) backbone were most frequently observed (n = 22). The antiretroviral drugs which were most frequently substituted for DRV were efavirenz (n = 8) and dolutegravir (n = 8). Four individuals did not reach the primary endpoint measurements as a result of gastro-intestinal side-effects (n = 2), loss to follow-up (n = 1), or because the additional study measurements were experienced as a burden (n = 1).

### 3.1. Virology

**Figure 1:**
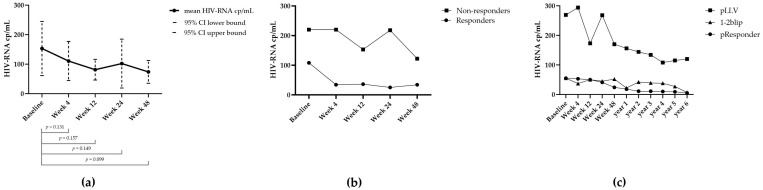
(**a**) Mean overall viral load dynamics with a 95% confidence interval at each time point. BL = 161 cp/mL; WK4 = 112 cp/mL; WK12 = 81 cp/mL; WK24 = 102 cp/mL; WK48 = 74 cp/mL. No significant differences were observed between the different time-points. (**b**) HIV-RNA dynamics (mean viral load) of responders and non-responders at different time-points. (**c**) HIV-RNA dynamics (mean viral load) of persistent responders, pLLV, and 1–2 blips at different time points during the study period and follow-up time.

The study population could be divided into responders (N = 16, 53%), non-responders (N = 12, 40%), or unassigned (2 individuals; L-13 and L-24) at week 24. L-13 stopped DRV on their own initiative, and L-24 had no follow-up data after the switch (Table 2). The HIV-RNA dynamics are shown in Figure 1a–c. Overall, no significant decrease in HIV-RNA levels was seen between the baseline and different time points (Figure 1a). The viral load at baseline (strata ≤ 50 cp/mL, 51–199 cp/mL, 200–399 cp/mL, and ≥400 cp/mL) is associated with the virological response. A low viral load at baseline is more likely to result in a viral load of <50 cp/mL at week 24 (*p* = 0.013). This association was, however, not seen when only strata 51–199 and 200–399 were taken (*p* = 0.25).

As part of a post-hoc analysis, the long-term response could be evaluated in 22 individuals (75% of the study population), with an average follow-up time of 4.7 years. Of these 22 individuals, 14 (63.6%) were correctly classified at week 24. Eight individuals switched response categories when compared to categorization at week 24. Only one individual who was a responder at week 24 had LLV during long-term follow-up, while three others had 1–2 blips. Three non-responders at week 24 eventually became responders during the long term-follow up, and one non-responder had 1 blip in the follow-up period. For more details, see Table 2.

Sequencing of reverse transcriptase and protease (pol) was performed in 22 individuals, and longitudinal sequence data were obtained for 9 individuals. There were no signs of evolution or selection of drug resistance in the protease or reverse transcriptase, although one individual had NNRTI mutations (K103N, Y188C) and a TAM (D67DN) at baseline (L-13). In two of the individuals, the viral population consisted partially of defective viruses. In individual L-2, several APOBEC-associated mutations and multiple stop codons (W88*, W212*, W266*) were detected in the reverse transcriptase at baseline, implying that viral production accounts for a significant part of the observed viremia, even though we found intact proviral DNA (13 cp/10^6^ PBMC) at baseline (week 24 could not be assessed). In individual L-22, the protease active-site mutation D25N was found to be the predominant viral population (±70%). The presence of this mutation still allows for dimer formation of protease, but the enzyme is unable to cleave viral proteins [29]. Intact proviral DNA was found at baseline (490 cp/10^6^ PBMC) and week 24 (66 cp/10^6^ PBMC) in L-22. Viral evolution was assessed for all individuals in which longitudinal sequence data were available (N = 9). Interestingly, the individual (L-2) for whom multiple stop codons and APOBEC-associated mutations were observed was the only individual for whom a significant change in the viral population occurred (dN-dS = 3, dN/dS = 0.8, *p* = 0.005), with hypermutation and not evolution as a proposed underlying mechanism for genetic change. In the other eight individuals (L-3, L-4, L-5, L-14, L-18, L-22, L-26, L-28), no relevant change in the viral population was observed using population sequencing.

### 3.2. Quantification and Activity of HIV Viral Reservoir

**Figure 2:**
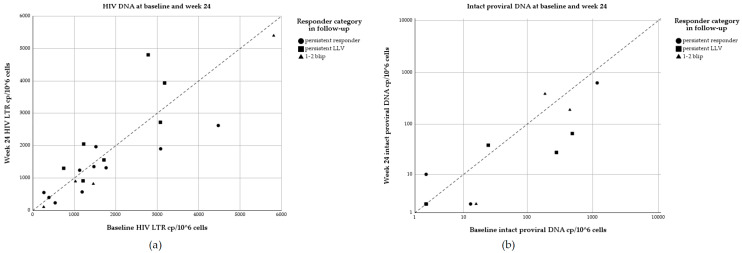
(**a**) Scatterplot of HIV-DNA dynamics at baseline and week 24 for the following categories: persistent responders (circle), persistent LLV (square), and individuals with 1–2 blips (triangle). No significant differences were seen in the HIV-DNA levels overall or in the subgroups. The diagonal dotted line represents an equal value at baseline and week 24. The values above the dotted line represent individuals with a higher value of HIV LTR cp/10^6^ cells at week 24. The values under the dotted line represent higher values of HIV LTR cp/10^6^ cells at baseline. (**b**) Intact proviral DNA is plotted at baseline and week 24 on a logarithmic scale. The area under the dotted line represents higher values of intact proviral DNA in cp/10^6^ cells at baseline, and the values above the dotted line represent a higher value at week 24.

To assess the size of the viral reservoir in PBMCs, HIV-DNA levels were measured at baseline and week 24. On a group level, no differences in LTR DNA levels were observed between baseline and week 24 (*p* = 0.882), nor were differences seen in the LTR DNA levels of responders (BL: 1466 cp/10^6^ cells; WK24: 1353 cp/10^6^ cells; *p* = 0.776) or non-responders (BL: 1245 cp/10^6^ cells; WK24: 1560 cp/10^6^ cells; *p* = 0.959) (Figure 2). When long-term follow-up was used for categorization, no differences were seen in the LTR DNA levels of persistent responders (BL: 1583 HIV LTR cp/10^6^ cells; WK24: 1216 HIV LTR cp/10^6^ cells), the persistent LLV group (BL: 1991 HIV LTR cp/10^6^ cells; WK24: 2470 HIV LTR cp/10^6^ cells) or the 1–2 blip group (BL: 2140 HIV LTR cp/10^6^ cells; WK24: 1805 HIV LTR cp/10^6^ cells) at baseline (*p* = 0.620) or week 24 (*p* = 0.131). 

**Figure 3:**
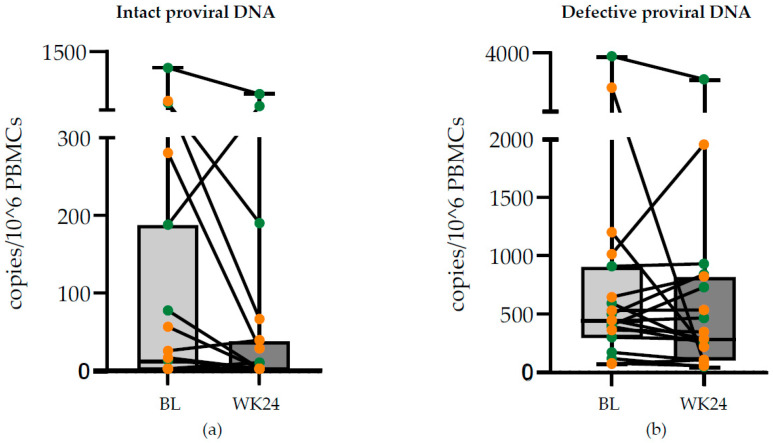
IPDA and defective proviral DNA at baseline and week 24. Boxplots of (**a**) total intact proviral DNA and (**b**) defective proviral DNA at baseline and week 24. Green dots are responders. Orange dots are non-responders. Lines represent changes within an individual. A trend was seen for lower intact proviral DNA at week 24 (*p* = 0.050), but not for the defective fraction (*p* = 0.398).

A trend towards lower levels of intact proviral DNA levels at week 24 compared to the baseline was observed (*p* = 0.050), possibly more pronounced in non-responders (*p* = 0.080) and not seen in responders (*p* = 0.249) (Figure 3a). This trend was not seen in the defective fractions of proviral DNA (*p* = 0.398) (Figure 3b).

Also, when long-term follow-up was used for categorization, no differences were seen in intact proviral DNA levels between the persistent responders, persistent LLV group, or the group with 1–2 blips at baseline (*p* = 0.243) or week 24 (*p* = 0.395). No significant differences were seen in msRNA when the baseline and week 24 were compared (*p* = 0.593), nor were differences observed in msRNA between persistent responders, persistent non-responders, or the group with 1–2 blips at baseline (*p* = 0.815) or week 24 (*p* = 0.762).

No correlation was found between the DNA LTR levels and intact proviral DNA at the baseline or week 24. A correlation was seen in both responders and non-responders with the defective fraction of the viral reservoir (psi+env) at baseline (r = 0.861; *p* = <0.01), but not at week 24. A correlation between DNA LTR levels and the defective fraction at baseline was observed both in the non-responders (r = 0.830, *p* < 0.01) and in the responders (r = 0.918, *p* < 0.01). The intact fractions of proviral DNA and msRNA were not correlated at baseline or week 24. msRNA was, however, correlated with the defective fraction at baseline (r = 0.46; *p* = 0.042) and at week 24 (r = 0.52, *p* = 0.047). msRNA and DNA LTR levels were also correlated at baseline (r = 0.47; *p* = 0.044), but not at week 24 (*p* = 0.242). In the non-responders, a correlation was seen at week 24 with the defective fraction with msRNA (r = 0.845; *p* = 0.034), which was not observed in the responders.

### 3.3. Pharmacology and Therapy Adherence

A once-daily ART regimen (DRV 800 mg) was used in 28 individuals, and 2 individuals used a twice-daily (DRV 600 mg) scheme. In one individual (L-13), DRV plasma concentrations were not detectable at week 4 or week 24. This individual had therapy failure at week 24. In three individuals, DRV drug levels were below the 1.07 mg/L (once daily) threshold at either week 4 (L-5) or week 24 (L-27, L-29). All the other individuals had levels of DRV above the lower limit of efficacy. See Supplementary S2 for DRV drug level measurements. On a group level, the DRV levels were not different between the responders and non-responders (week 4, *p* = 0.894; or week 24, *p* = 0.461). When long-term follow-up was used for categorization, no differences were seen in the DRV levels between persistent responders, persistent LLV, or individuals with 1–2 blips (week 4, *p* = 0.906; week 24, *p* = 0.232). The mean self-reported adherence was 90% or higher in all the individuals. Self-reported adherence was associated with DRV drug levels (*p* < 0.01). One individual who reported non-adherence (L-13) also had no detectable DRV levels at week 4 and week 24. A total of 25 individuals who had high self-reported adherence (mean > 99%) also had detectable drug levels. Only in three individuals, L-5, L-27, and L-29, was self-reported adherence high, but the DRV concentration was below 1.07 mg/L at either week 4 or 24. During long-term follow-up, 12 out of the 22 individuals (55%) still used boosted DRV.

### 3.4. Immune Activation

#### 3.4.1. Soluble Markers

Ten soluble immune markers were compared at baseline and week 24. Overall, significant differences were seen for IL-1b (*p* = 0.013) and MIP-1b (*p* = 0.014), with lower levels of these markers at baseline (median IL-1b = 1.19 pg/mL; MIP-1b = 70.97 pg/mL) compared to week 24 (IL-1b = 2.06 pg/mL; MIP-1b = 141.20 pg/mL). These differences were not significant when a Bonferroni correction for multiple testing was applied (level of significance at 0.005). No significant differences for other markers were seen on a group level.

Within the persistent responder group, an increase of IL-1b (BL = 1.32 pg/mL; WK24 = 3.84 pg/mL (*p* = 0.021)) and MIP-1b (BL = 65.49 pg/mL; WK24 = 168.35 pg/mL (*p* = 0.018) was seen. In contrast, MIG levels (BL = 28.25 pg/mL; WK24 = 20.06 pg/mL) decreased over time (*p* = 0.018). These differences were, however, not significant when a Bonferroni correction for multiple testing was applied. IP-10 had a low correlation, with HIV-DNA at WK24 only (r = 0.69; *p* < 0.05)). No other correlations between soluble markers and virological characteristic were observed. See Supplementary S3 and S4 for measurements of all immunological markers.

#### 3.4.2. Cell-Bound Markers

When comparing subsets of activated cells at baseline and week 24, we found that activated (CD38^+^HLA-DR^+^) CD4^+^ memory T cells were higher at baseline than week 24 (median = 3.37 at baseline and median = 2.64 at week 24, *p* = 0.041), but not for activated CD4^+^ total CD38^+^HLA-DR^+^, CD8^+^ total CD38^+^HLA-DR^+^, or CD8^+^ memory CD38^+^HLA-DR^+^ cells). No significant differences were seen between the responders and non-responders in any of the CD38^+^HLA-DR^+^ T cells (CD4^+^ total, CD4^+^ memory, CD8^+^ total, CD8^+^ memory) at baseline or week 24. The HIV-DNA levels at week 24 were moderately correlated with the total activated CD4^+^ (r = 0.68; *p* < 0.05) and activated CD4 memory subset (r = 0.66; *p* < 0.05)), but not with activated CD8^+^ cells nor with HIV-DNA at baseline. No correlation was found between msRNA, intact proviral DNA, or defective proviral DNA with any of the CD38^+^HLA-DR^+^ T cells. See Supplementary S3 and S4 for measurements of all immunological markers.

## 4. Discussion

In this study, we investigated the dynamics of HIV-RNA and immune markers after switching to DRV in people with LLV. We hypothesized that by switching to DRV, viral suppression would be observed in patients in whom LLV occurred due to continuous viral replication. If viral suppression would not occur, the viremia could be attributed either to viral production or low-level replication from sanctuary sites.

We observed a response (viral suppression) in 16 individuals (53%) in our study within 12 weeks of the switch. This makes (treatable) viral replication a suggestive source of LLV in approximately half of our study population. Several clinical studies of therapy intensification have been performed in individuals on effective ART to differentiate between active replication and viral production as a source of residual viremia. However, these studies were performed in individuals with residual viremia (detectable viral loads below the cut-off of 50 copies/mL), and they found no evidence of replication [30,31,32,33]. In a small study of individuals experiencing LLV (50–1000 copies/mL) during ART, viral suppression was obtained after switching to a DRV/r based regimen [34]. In this study, protease inhibitors were replaced by DRV, and the population had a much higher baseline viral load (774 cp/mL) compared to our study (153 cp/mL). At week 24, they observed viral suppression in 93% of the individuals, with a relatively high-range LLV after the switch, suggesting responsiveness to therapy and suppression of viral replication.

Our study, which had a lower range of viremia, found a lower rate of responsiveness to the switch. Of interest, in 63.6% of those who responded at week 24, a long-term response was observed at follow-up. Only one individual who was a responder at week 24 did return to persistent LLV during follow-up. Hence, for insight into the dynamics of LLV, a 24-week observation period can already provide a useful indication.

We hypothesized that if replication discontinued, one might see lower levels of intact proviral DNA at week 24 in the responder group. Although we did observe a trend towards lower levels of intact proviral DNA at week 24, this was not significant and was not explicitly seen in the responders. The most likely explanation is that discontinuation of low levels of viral replication only marginally affect the size of the total intact proviral DNA reservoir, and/or part of the LLV is originating from infected cells in the tissue.

It cannot be excluded that in those with a sudden undetectable HIV-RNA at baseline improved their adherence, which may have resulted in persistent viral suppression. A decrease in HIV-RNA after the switch to darunavir could theoretically also be the result of ceased viral production due to cell death, senescence of HIV-producing T lymphocytes, or improved adherence by taking part in this study. The variability in detectable HIV-RNA levels might also be explained by homeostatic proliferation [35] and the size of the HIV reservoir [22]. Of interest is that correlations with the defective fraction were seen for HIV LTR DNA and msRNA in the non-responders, possibly reflecting the HIV-RNA or viral production by defective proviruses seen by others [36].

We observed that, in a subset of individuals, LLV persisted, despite starting with a high genetic barrier protease inhibitor-containing regimen. Most of these individuals had LLV in a relatively low range (<400 cp/mL). A possible explanation could be that there was an insufficient effect of the switch to a boosted DRV-containing regimen in anatomical or physiological viral reservoirs, such as the central nervous system, lymphoid tissue, or the gut [37], but this could not be investigated thoroughly in our study. In one subject (L-28), a non-responder, we had CSF available at baseline and week 24, but this person had an undetectable HIV viral load in CSF at both time points (see Supplementary S5). To evaluate whether replication was still occurring, we evaluated whether viral evolution could be observed in the longitudinal sequence data of reverse transcriptase and protease in a subset of nine individuals, but we did not find any evidence for viral evolution. In one individual, several mutations and changes were seen, but this was considered to be the result of (APOBEC) hypermutation instead of evolution. Other research groups have also shown that evolution is not always observed in individuals with LLV [10], and that persistent LLV can come from large cell clones carrying (non-)intact proviruses [38] that can produce RNA and non-infectious virions [39]. For this reason, in the absence of viral evolution, we consider replication to be an unlikely source of persistent LLV in our cohort of individuals who switched to a boosted DRV-containing regimen and were actively monitored.

Viral blips and LLV might be associated with reservoir size [22,40]. For this reason, we were interested in determining whether was a difference in the size of the total reservoir as well as the fraction of intact proviral reservoir in the responders and non-responders, but this was not seen in our study. Of interest is that in viral production of both intact and defective HIV RNA can circulate, which is supported by our data from RT and PR genotyping in L-2 and L-22, which showed that the viral RNA consisted partly of circulating defective viral populations.

An HIV infection, even when properly treated, is associated with higher levels of immune activation compared to people living without HIV [41]. In previous studies, individuals with undetectable viremia had lower levels of sCD14 than individuals with residual or LLV [42]. In our study, switching to a DRV-containing regimen did not result in lower levels of immune markers. Even though we observed some suggestive, albeit contrasting, findings like higher levels of MIP-1b and IL-1b at week 24 and lower levels activated CD38+ HLA-DR+CD4 T cell memory cells at week 24 compared to the baseline, these findings were not significant when we corrected for multiple testing. In our relatively small sample size, no correlations or significant differences were seen between immunological or viral reservoir parameters at baseline or week 24 when comparing the responders and non-responders.

Poor therapy adherence should always be considered as a potential reason for LLV [43,44]. In this study, individuals with presumed inadequate therapy adherence were excluded, and self-reported adherence in this study was high. DRV drug levels were associated with self-reported adherence, and adequate adherence was confirmed in the great majority of individuals, with non-adherence reported in one individual.

This study presents a unique data set of 30 individuals with LLV from different sites in the Netherlands with detailed information about HIV-RNA dynamics, the size of the (intact) HIV reservoir, longitudinal genetic characterization of circulating HIV-RNA in the blood, and several soluble and cell-bound immunological parameters. This study does, however, have some limitations. As it is an observational study without a control group, we cannot compare our findings with HIV-negative controls, fully HIV-suppressed individuals, or those with high levels of circulating HIV-RNA. Additionally, the small sample size does not allow us to draw definite statistical conclusions regarding certain observations, and the low levels of HIV RNA make it difficult to accurately define quasispecies dynamics. Nevertheless, this study provides valuable in-depth insights in individuals with LLV despite good therapy adherence.

## 5. Conclusions

Unexplained LLV despite sufficient adherence, adequate therapy, and frequent follow-up can be the consequence of both viral replication and production. In approximately half of the study population, a switch to a DRV-based regimen resulted in a VL response within 24 weeks, indicative of ongoing low-level viral replication as the cause of LLV before the switch. In the non-responders, no signs of viral evolution or selection of drug resistance after switching to DRV was observed, suggestive of production. In a subset of the individuals with persistent LLV, production of defective HIV contributed to LLV. Our data confirm that in clinical management, high genetic barrier drugs like DRV are a safe choice, irrespective of the source of LLV.

## Figures and Tables

**Table 1:** Baseline characteristics.

Baseline Characteristics	N = 30
Mean age (σ)	49.4 (8.4)
Sex (male)	86.7%
Mean viral load (σ)	153 cp/mL (246)
Mean CD4 count (σ)	667 cells/mm^3^ (364)
HIV subtype	B:20; C:2; CRF02_AG:2; F:2; A:1; unknown: 3
Mean intact proviral DNA (σ)	172 cp/10^6^ cells (293)
Mean msRNA (σ)	11.9 cp/µg RNA (26.1)
Mean LTR-DNA (σ)	1754 cp/10^6^ cells (1333)
ART at switch	TDF, FTC, **EFV**: 6 TDF, FTC, **DTG**: 3 TAF, FTC, **DTG**: 1 TAF, FTC, **EVG/c**: 3 TDF, FTC, **ATV/r**: 2 TDF, FTC, **NVP**: 2 ABC, 3TC, **NVP**: 2 ABC, 3TC, **DTG**: 2 TDF, FTC, **RAL**: 1 TDF, FTC, **RIL**: 1 TAF, FTC, **RIL**:1 TDF, 3TC, **EFV**: 1 ABC, 3TC, **LPV/r**: 1 ABC, 3TC, **EFV**: 1 TDF, DTG, **ATV/r**: 1 TDF, FTC, DTG, MVC, **LPV/r**: 1
Substance use	Nicotine 13 (43.3%) Alcohol > 2 per day: 2 (6.7%) Cannabis: 7 (23.3%)
Co-morbidities	DM: 0% CVD: 3 (10%)
	COPD: 3 (10%)

ABC (abacavir), TDF (tenofovir disoproxil), TAF (tenofovir alafenamide), EFV (efavirenz), DTG (dolutegravir), NVP (nevirapine), EVG/c (boosted elvitegravir), RIL (rilpivirine), ATV/r (boosted atazanavir), LPV/r (boosted lopinavir), RAL (raltegravir), MVC (maraviroc), DM (diabetes mellitus), CVD (cardiovascular disease), COPD (chronic obstructive pulmonary disease). σ = standard deviation. In bold: old anchor before switch.

**Table 2:** HIV RNA and DNA dynamics on an individual level categorized by responder group. The upper part shows responders at week 24. The middle part shows non-responders at week 24. The lower part shows the unassigned individuals. Follow-up response category after week 48 is defined as either persistent responder (none of the measurements > 50 cp/mL), 1–2 blips, persistent low-level viremia (pLLV) including how many of the measurements (in %) were >50 cp/mL in the follow-up period, or no follow-up available. HIV-DNA: HIV LTR cp/10^6^ cells. IPDA: intact proviral DNA in cp/10^6^ cells. msRNA = multiply spliced RNA in copies/µgRNA. HIV-RNA loads are depicted for screening, baseline (BL), week 4 (WK4), week 12 (WK12), week 24 (WK24), and WK 48 (WK48). Viral loads > 50 cp/mL are depicted with a light gray background. Short-term response at week 24 is defined as responder (R), non-responder (NR), or unassigned (-). O = response category in follow-up other than week 24; “=” equal response category in follow-up to week 24.

	HIV-DNA		IPDA (Intact)		msRNA			HIV-RNA						Short Term (WK24)	Follow-Up		
	BL	WK24	BL	WK24	BL	WK24		SCR	BL	WK4	WK12	WK24	WK48	Responder (R) Non-Responder (NR)	% Measurements >50 cp/mL		Time (yrs)
L-1	1767	1317	1.96	1.96	0	0		80	0	40	40	20	0	R	persistent responder (0%)	=	6.3
L-7	3083	1903	1171	630	0	0		46	46	<40	<40	<40	65	R	persistent responder (0%)	=	4.6
L-12	1522	1968	-	1.96	-	32		77	<40	80	<40	<40	<40	R	persistent responder (0%)	=	5.2
L-16	388	400	1.96	10	0	0		75	0	48	40	48	<40	R	persistent responder (0%)	=	5.9
L-17	538	234	13	1.96	0	0		44	0	0	0	0	0	R	persistent responder (0%)	=	5.3
L-19	1473	1353	-	-	-	-		50	50	<20	37	<20	<20	R	persistent responder (0%)	=	5.5
L-21	1189	571	-	-	-	-		83	112	50	<40	<40	<40	R	persistent responder (0%)	=	4.9
L-29	266	553	1.96	1.96	-	0		53	53	0	<40	0	<40	R	persistent responder (0%)	=	3.5
L-10	1459	821	450	190	0	0		52	76	62	<40	40	53	R	2 blip (<25%)	O	5.3
L-15	5811	5397	188	387	64	-		75	<40	<40	<40	<40	<40	R	1 blip (<25%)	O	5.9
L-30	1028	896	1.96	1.96	0	0		41	41	0	<40	0	<40	R	2 blip (<25%)	O	3.2
L-27	2783	4806	1.96	1.96	14	-		56	56	17	<40	<40	100	R	pLLV 53%	O	3.4
L-6	2794	2665	-	-	-	-		66	0	55	40	0	-	R	no longitudinal follow-up	.	-
L-9	924	3836	-	35	-	0		125	1010	0	<40	40	-	R	no longitudinal follow-up	.	-
L-23	731	1410	77	1.96	0	-		151	146	57	18	0	0	R	no longitudinal follow-up	.	-
L-25	2617	-	715	-	0	-		51	51	<40	-	-	-	R	no longitudinal follow-up	.	-
L-2	3077	2722	13	-	0	0		189	219	176	62	144	64	NR	pLLV 100%	=	5.3
L-3	3179	3935	281	28	23	21		189	1020	487	413	1010	348	NR	pLLV 82%	=	7.0
L-18	1226	2051	1.96	1.96	0	0		168	168	842	202	83	231	NR	pLLV 75%	=	4.2
L-22	1211	914	490	66	0	0		200	93	270	128	167	88	NR	pLLV 100%	=	4.2
L-26	1715	1560	-	-	-	-		268	268	228	205	300	266	NR	pLLV 100%	=	3.3
L-28	744	1302	25	39	30	-		70	58	<40	162	135	90	NR	pLLV 63%	=	3.6
L-14	261	106	16	1.96	-	-		60	63	44	80	100	73	NR	1 blip (<25%)	O	6.5
L-4	1129	1242	1.96	1.96	6	15		46	42	100	100	100	40	NR	persistent responder (0%)	O	2.1
L-5	-	-	1.96	1.96	0	0		172	172	170	138	92	<20	NR	persistent responder (0%)	O	6.3
L-11	4474	2623	1.96	1.96	100	0		133	93	40	<40	52	0	NR	persistent responder (0%)	O	5.1
L-8	1265	-	133	-	0	-		272	272	75	-	-	-	NR	no longitudinal follow-up	.	-
L-20	149	289	56	1.96	0	-		110	170	170	-	-	-	NR	no longitudinal follow-up	.	-
L-13	-	10080	-	-	-	-		166	166	20	50	228,000	-	-	no longitudinal follow-up	.	-
L-24	2314	-	313	-	-	-		69	69	-	-	-	-	-	no longitudinal follow-up	.	-
							N	30	30	29	26	26	23				

## Data Availability

The authors confirm that the data supporting the findings of this study are available within the article. Further inquiries can be directed to the corresponding author.

## Supplementary Materials

The following supporting information can be downloaded at: https://www.mdpi.com/article/10.3390/v16020182/s1, S1: msRNA primers and probes; S2: Pharmacology; S3: soluble markers; S4: cell associated markers; S5: HIV-RNA in CSF-Plasma
