# Supplementary material for: Dynamics of Low-Level Viremia and Immune Activation after Switching to a Darunavir-Based Regimen
Source: Viruses. 2024 Jan 25;16(2):182. doi: 10.3390/v16020182 (PMC10893305; doi:10.3390/v16020182)
Supplement: Supplementary file 1 [file viruses-16-00182-s001.zip › Supplementary S1_msRNA primers and probes_def.pdf]

subtype	name	sequence	position relative to HXB2
B	msRNA Tat 1.4	5' TGGCAGGAAGAAGCGGAGA 3'	5971-5989
	msRNA-B-FAM/ZEN	5'-56-FAM/TTCCTTCGG/ZEN/GCCTGTCGGGT/3IAKbFQ/-3'	8402-8421
	msRNA-B-rev	5' GGATCTGTCTCTGTCTCTCTCTCCACC 3'	8433-8459
C	msRNA C forw-2c	5' GCAGTRAGGATCATCAARATCYTRTATCAAAGC 3'	6012-6044
	msRNA -C-FAM/ZEN	5'-/56-FAM/CTTCTTCGA/ZEN/TTCYTCCGRGCCTGTC/3IABkFQ/-3'	8406-8430
	msRNA -C-rev	5'GATCTGYCTYTGTCTTGCTCTCCACCT3'	8432-8458
