# Supplementary material for: Dynamics of Low-Level Viremia and Immune Activation after Switching to a Darunavir-Based Regimen
Source: Viruses. 2024 Jan 25;16(2):182. doi: 10.3390/v16020182 (PMC10893305; doi:10.3390/v16020182)
Supplement: Supplementary file 1 [file viruses-16-00182-s001.zip › Supplementary S2_Pharmacology.pdf]

Supplementary S2. DRV drug levels at week 4 and week 24

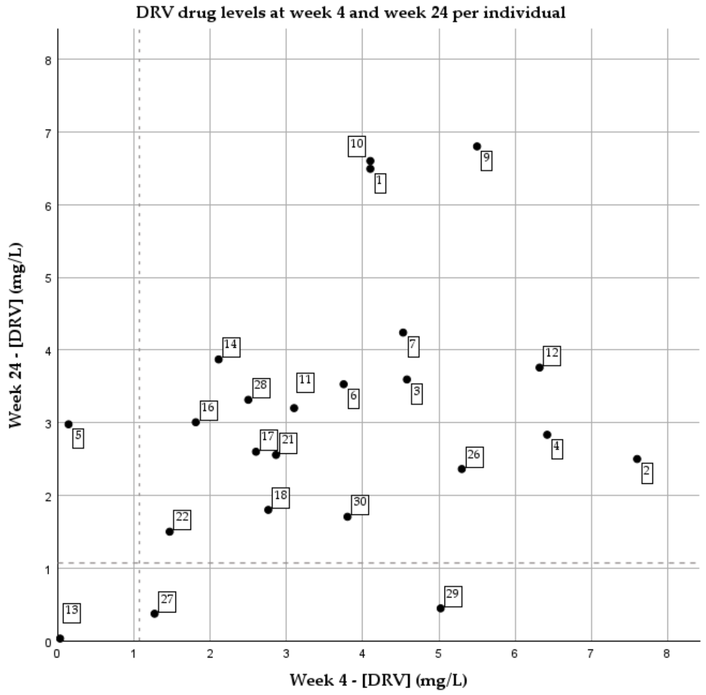

DRV drug levels (mg/L) per individual at week 4 and week 24. Reference level for once daily use (1.07 mg/L) is depicted as a dashed line. Non-adherence is seen in L-13 at week 4 and 24. In three individuals drug levels below reference through levels (1.07 mg/L) at either week 4 (L-5) or week 24 (L-27, L-29). Potential explanations for levels below 1.07 mg/L include suboptimal adherence, drug-drug interactions or (temporary) gastro-intestinal symptoms like diarrhea. Individuals with only DRV drug levels at week 4 are not depicted in this figure: L-8 (9.14 mg/L) , L-20 (2.94 mg/L), L-25 (1.95 mg/L). Individuals with only DRV drug levels at week 24 are also not depicted in this figure L-15 (2.9 mg/L), L-19 (2.0 mg/L), L-23 (4.3 mg/L).
