# Supplementary material for: Dynamics of Low-Level Viremia and Immune Activation after Switching to a Darunavir-Based Regimen
Source: Viruses. 2024 Jan 25;16(2):182. doi: 10.3390/v16020182 (PMC10893305; doi:10.3390/v16020182)
Supplement: Supplementary file 1 [file viruses-16-00182-s001.zip › Supplementary S5_CSF-Plasma.pdf]

Supplementary S5: HIV-RNA in CSF – Plasma

Subject ID	Baseline (plasma)	Baseline (CSF)	Week 24 (plasma)	Week 24 (CSF)
**L-15**	**<40 cp/mL**	**0 cp/mL (TND)**	**<40 cp/mL**	**n.a.**
**L-28**	**58 cp/mL**	**<50 cp/mL**	**135 cp/mL**	**0 cp/mL (TND)**

n.a. = not available; TND = Target not detected; CSF = cerebrospinal fluid
