# Supplementary figures and images for: Dynamics of Low-Level Viremia and Immune Activation after Switching to a Darunavir-Based Regimen
Source: Viruses. 2024 Jan 25;16(2):182. doi: 10.3390/v16020182 (PMC10893305; doi:10.3390/v16020182)

**IL-1b**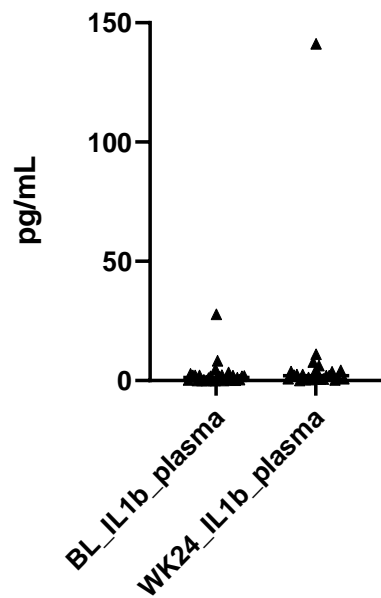**IL-6**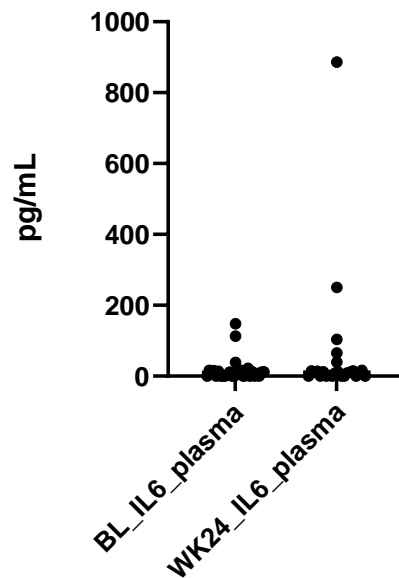**MIP-1a**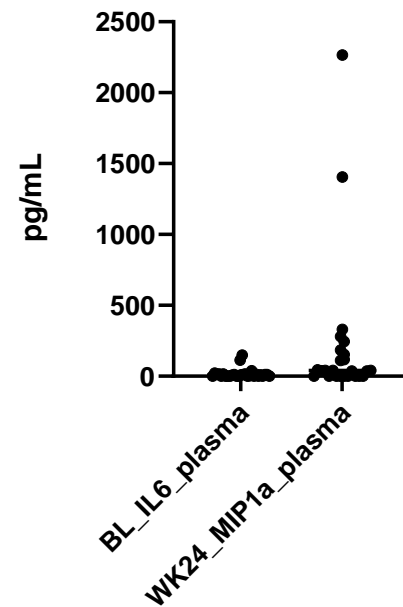**MIP1b**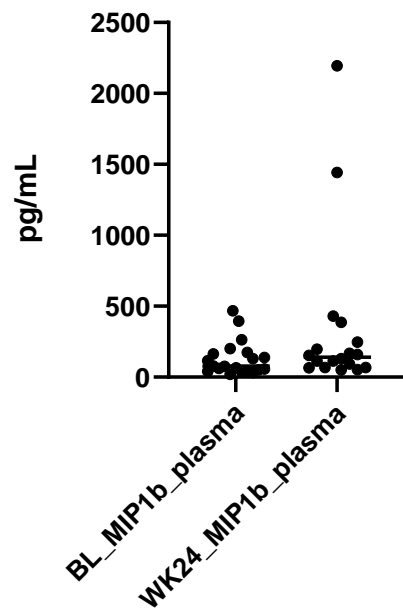**MCP1**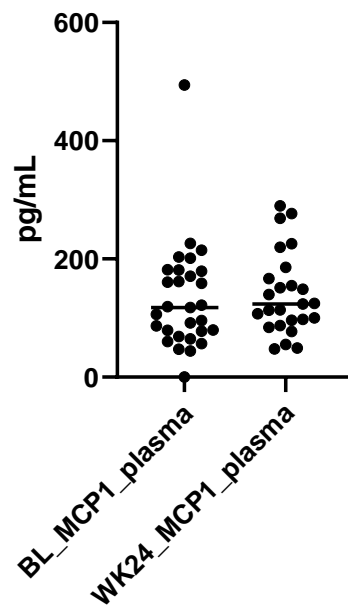**IP-10**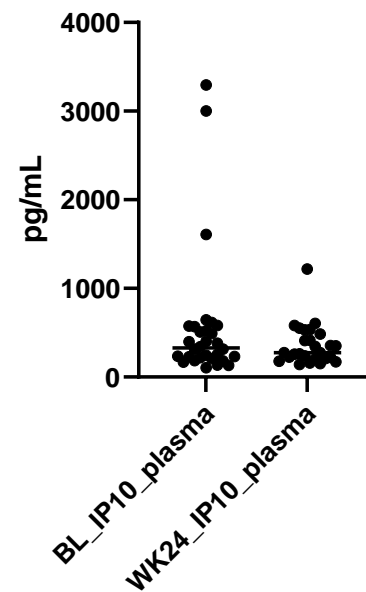

Supplement: Supplementary file 1 [file viruses-16-00182-s001.zip › Supplementary S3a_soluble markers.pdf]

**sICAM (log)**

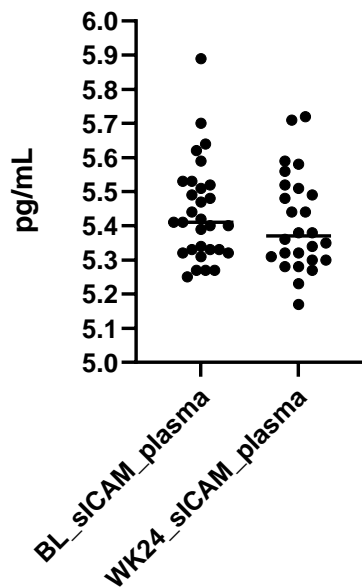

**sCD14 (log)**

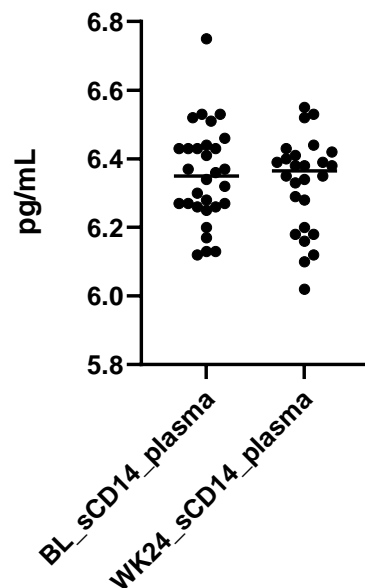

**sCD163**

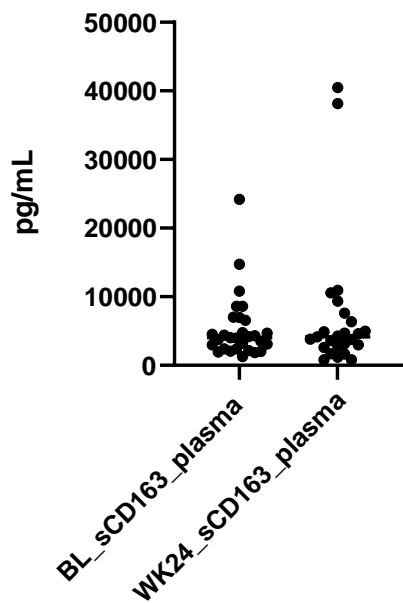

**MIG**

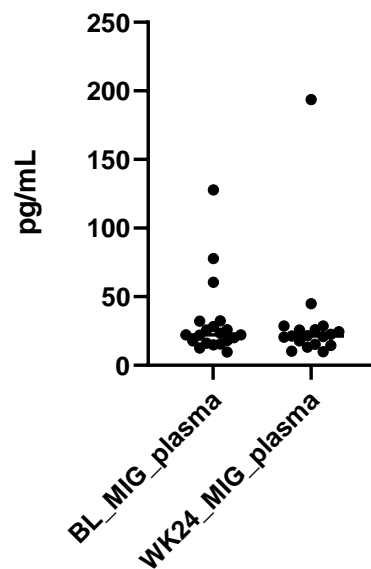

Supplement: Supplementary file 1 [file viruses-16-00182-s001.zip › Supplementary S3b_soluble markers.pdf]

**CD4+CD38+HLA-DR+ (total)**

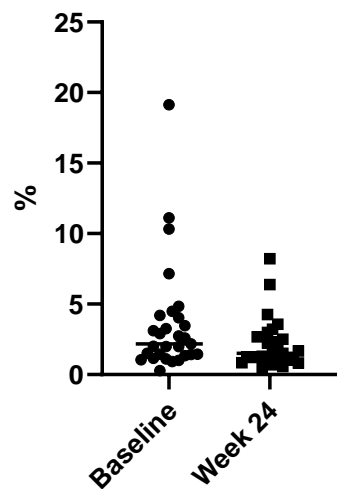

**CD4+CD38+HLA-DR+ (memory)**

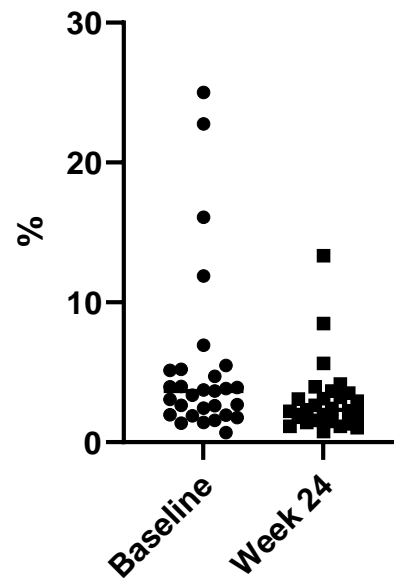

**CD8+CD38+HLA-DR+ (total)**

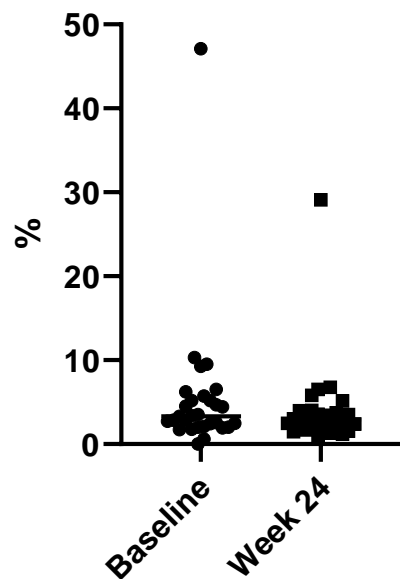

**CD8+CD38+HLA-DR+ (memory)**

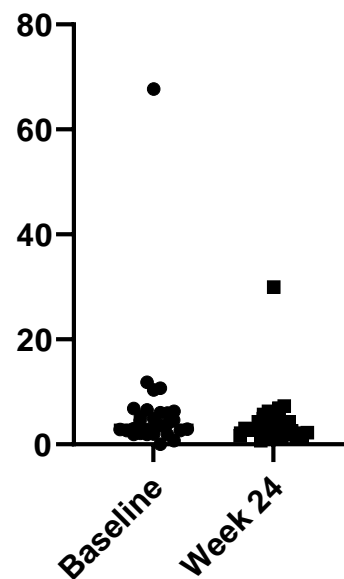

Supplement: Supplementary file 1 [file viruses-16-00182-s001.zip › Supplementary S4_cell assiociated markers.pdf]
